# Some common statistical methods for assessing rater agreement in radiological studies

**DOI:** 10.1177/02841851251319666

**Published:** 2025-02-23

**Authors:** Mats Geijer, Magnus Båth, Catrin Wessman

**Affiliations:** 1 Department of Radiology, Institute of Clinical Sciences, 70712 Sahlgrenska Academy, University of Gothenburg, Gothenburg, Sweden; 2 Department of Radiology, Region Västra Götaland, Sahlgrenska University Hospital, Gothenburg, Sweden; 3 Department of Clinical Sciences, 5193 Lund University, Lund, Sweden; 4 Department of Medical Radiation Sciences, Institute of Clinical Sciences, 70712 Sahlgrenska Academy, University of Gothenburg, Gothenburg, Sweden; 5 Department of Medical Physics and Biomedical Engineering, Sahlgrenska University Hospital, Gothenburg, Sweden; 6 Biostatistics, School of Public Health and Community Medicine, Institute of Medicine, 70712 Sahlgrenska Academy, University of Gothenburg, Gothenburg, Sweden

**Keywords:** Statistical methods, observer agreement, observer variation, radiological studies, image quality assessment

## Abstract

Rater agreement is commonly assessed in radiologic studies concerning image quality. There are several methods of measuring rater agreement. To choose the appropriate method, the researcher needs to consider the scale of the outcome variable and the design of the study. This article provides a brief overview of available methods, focusing on the most practical and commonly used, including intraclass correlation, the Svensson method, variants of the kappa statistic, the agreement coefficient by Gwet (AC1/AC2), and Krippendorff's alpha. Additional methods that are not primarily intended for rater agreement analysis but are applied in some cases are also discussed.

## Background

The subjective interpretation of radiologic images and the existence of large variations between and within raters have been documented since the 1940s (1,2). Rater agreement remains an important issue in the analysis of subjective rater data in radiologic studies where the variation between raters may be greater than between images (3).

Radiology is the field where rater agreement is most studied because reviewing images is easier than evaluating clinical findings on patients by multiple evaluators or in repeated assessments. Rater agreement has, however, been analyzed, albeit more seldomly, in other clinical areas. Yet, rater agreement or disagreement is rarely included as a bias in scientific studies or reported as a limitation outside radiologic research.

There are several methods to measure rater agreement, both between separate raters (inter-rater reliability) and within the same rater in repeated observations (intra-rater reliability). Terminology may differ; terms and synonyms are listed in Table 1. Hayes and Krippendorff (4) compared several statistical methods to evaluate rater reliability, such as percent agreement, Bennett et al.'s score, Scott's π, Cohen's kappa, Fleiss’ kappa, Cronbach's alpha, and Krippendorff's alpha. The authors defined five criteria for the properties of a well-functioning index of reliability (4). In this article, we will discuss some of these methods from a radiological perspective and present additional methods, e.g. intraclass correlation (ICC) (5–7), the agreement coefficient by Gwet (AC1/AC2) (8–10), and the Svensson method (11,12). We will also comment on the suitability of other methods not primarily developed for but commonly used in rater agreement analysis. These include visual grading characteristics (VGC) analysis (13,14), visual grading regression (VGR) (15), consensus scoring (16), percent agreement (4), Pearson correlation (17), and the Bland–Altman plot (17).

**Table 1.** Definitions and synonyms of statistical terms used.

Definition	Synonyms and comments
Agreement	The amount with which two measurements coincide. Agreement parameters are expressed on the actual scale of measurement and not as a dimensionless value between 0 and 1 (18)
Estimate	The calculated value from a statistical method. Examples are mean value, *P* value, or kappa value
Indicator of agreement	Statistical method for rater agreement analysis
Inter	Relating to reliability between raters
Inter-rater reliability	The degree of agreement among independent raters who score the same phenomenon. Synonyms may be inter-rater agreement, inter-rater concordance, inter-observer reliability, inter-coder reliability, and inter-observer agreement
Intra	Relating to reliability within the same rater
Item	The individual unit of scoring. Synonyms may be individual, subject, object, or case
Outcome	The measured value to which the statistical method is applied. Synonyms may be a response, a measure, or an assessment. The synonym score applies when the outcome belongs to an ordinal scale; a piece of information, usually a letter or a number that conveys the subjectively perceived image quality
Rater	The human evaluator who subjectively judges the image quality. Synonyms may be observer, reader, or coder
Reliability	The overall consistency of a judgment. An assessment instrument is said to have high reliability if it produces similar results under consistent conditions. With a high reliability, there is a high agreement between raters and low variation. Reliability parameters are usually expressed as a dimensionless value between 0 and 1
Validity	Describes a test's ability to produce results consistent with other measures of the same characteristic and requires external criteria (19)

The issue of rater agreement analysis is complex, and no indicator of agreement is perfect, with different pros and cons. In addition, there is no direct connection between the estimate and the clinical utility, where a certain reported agreement can be sufficient in one setting but not in another. For a more robust assessment, the researcher may report two or more indicators, which should point in the same direction.

## Basic facts and definitions

Validity relates to how well the measurements mirror the population, i.e. whether we have a bias in our measurements or not (19). Agreement refers to how well one set of measurements agrees with another set of measurements of the same item. Reliability is a function of the variability, hence, how consistent the sample measurements are and how well one case can be distinguished from another (18). Validity and reliability are two basic concepts in analyzing rater agreement, but when studying rater agreement in image quality studies, validity is often hard to assess when there is no reference standard to compare with. Often, the study must assess subjective grading of image quality, where two or more scores are compared, thus focusing on reliability. An effort to obtain better external validity may be undertaken by including raters of different experience, better representing the general population of raters than only using expert raters.

Variations in given scores between raters may be due to differences in how they interpret the scale steps of the rating scale and the actual differences in the perceived image quality. To determine rater agreement using the methods discussed in the current article in a meaningful way, it must be assumed that raters interpret the rating scale in the same way so that a difference in given scores corresponds to an actual difference in perceived image quality. The raters’ subjective use of scale criteria adds to the variability in scoring. Methods for determining rater agreement when this assumption cannot be made are generally outside the scope of the current article. The VGC method (13) is an example of how the raters’ individual scores are of less importance and the method instead analyzes the size and direction of change in scoring.

## Criteria for method selection

To quantify rater agreement, different statistical approaches are required depending on the study design, measured outcomes, and data types (Tables 2 and 3). For example, the same rater might score the same image on different occasions, multiple raters may score the same image, or the same rater may score different aspects of image quality. The observations may be paired or repeated. The most appropriate statistical method depends on the scale of the outcome and differs for nominal, ordinal, or quantitative outcomes (Table 4).

**Table 2.** Different types of data.

Data type	Content	Real-world example
Categorical data	Characteristics of an observation. Further subdivided into binary, nominal, and ordinal data	
Binary	One of two possible states	Fracture present/not present
Nominal	Characteristics of an observation without order or rank between categories	Image type (ultrasound, computed tomography, radiograph)
Ordinal	Characteristics of an observation with a meaningful order or rank between categories. The size of the difference between the categories is unknown	Image noise level (low, medium, high) Image quality grading (A, B, C, D, E from bad to good)
Quantitative data	Numerical measurements of quantitative data. Further subdivided into interval and ratio data, both of which can be discrete or continuous	
Interval	Ordered quantitative data with equal spaces between measuring points. The zero point is arbitrary	Change in body weight Fahrenheit temperature
Ratio	Ordered quantitative data with equal spaces between measuring points. The zero point represents that there is nothing of the variable to measure	Length Time Count Kelvin temperature

**Table 3.** Levels of measurement for different data types.

	Nominal	Ordinal	Interval	Ratio
Categories	Yes	Yes	Yes	Yes
Mode	Yes	Yes	Yes	Yes
Order/rank		Yes	Yes	Yes
Median		Yes	Yes	Yes
Equal spacing			Yes	Yes
Arithmetic mean			Yes	Yes
Add and subtract			Yes	Yes
True absolute zero				Yes
Multiply and divide				Yes
Geometric mean				Yes

**Table 4.** Statistical methods for rater agreement analysis for various data types and rater numbers.

Data type	No. of raters	Possible (suggested) statistical method
Binary	2	Cohen's kappa
Binary	≥2	Multirater kappa, ICC, Fleiss’ kappa
Nominal	2	Cohen's kappa
Nominal	≥2	ICC, Krippendorff's alpha, Gwet's AC1
Ordinal or discrete	2	VGC, Cohen's kappa, the Svensson method
Ordinal or discrete	≥2	ICC, Krippendorff's alpha, Gwet's AC2
Continuous	2	Bland–Altman plot*
Continuous	≥2	ICC, Krippendorff's alpha, Gwet's AC2

*The Bland–Altman plot is a descriptive graphical tool.

ICC, intraclass correlation coefficient.

Ordinal data are common in radiologic image quality studies. These data result from scoring images on a ranking scale from worse to better and require special handling. With ordinal data, it is not meaningful to calculate a mean. Instead, reporting is limited to the mode and the median (Table 3). To present the counts of different scores visually, a frequency table or a frequency diagram, such as a heat map, may be used.

## Methods for evaluating rater agreement

### Intraclass correlation (ICC)

Characteristics of ICC include the following: data type = qualitative or quantitative; number of raters: more than two; analysis software = standard statistical packages; estimate to report = number between 0 and 1; significance analysis = 95% confidence interval (CI), *P* value; pros = handles a wide range of raters and observations; cons = may be difficult to understand and prone to statistical errors if not handled correctly.

ICC is a measure of agreement between raters on both quantitative and qualitative data within a class of data, such as magnetic resonance (MR) image quality at different field strengths, but not between different classes (e.g. field strength and echo time) (5). ICC should not be applied to small datasets, and it has been suggested that a heterogeneous sample of at least 30 observations and at least three raters should be included in the analysis (7). There are several ways to express ICC depending on whether the outcome is binary, ordinal, or continuous (6). Different adjustments for covariates can also be modeled. This makes the method very flexible regarding different study designs. It also makes ICC rather complex to calculate and the measures of correlation differ depending on which model is used. The researcher should answer four questions, the answers of which will guide the selection of the appropriate ICC model (6,7). First, will the same raters score all objects? If so, a two-way model is appropriate, otherwise a one-way model should be chosen. Second, are the raters selected from a larger population or a specific sample of raters? In the first case, a random-effects model may be used, otherwise a fixed-model should be used. Third, should the reliability of a single rater or the mean value of multiple raters be measured? In the first case, single-measure ICC must be used, otherwise average-measures ICC is appropriate. If single-measure ICC is low but average-measures ICC high, both can be reported to illustrate the discrepancy (20). Fourth, is consistency or agreement most important? If an ordinal scale is used, one rater may consistently score lower than another rater, but the ranking of scores between objects is similar and consistency is most important. If scores in absolute values need to be compared, agreement is most important. These important basic settings are vital to consider (e.g. which model is used) when calculating ICC. Variations of the settings will give different results (7). It is vital to understand these settings before calculating, and it is also very important to report the settings. Otherwise, the reported ICC results will be impossible to understand.

An extensive section describing how to calculate ICC may be found in Gwet 2014 (10). Liljequist et al. (5) suggest a slightly different method for choosing the correct settings than the verbal recommendations by Koo et al. (7) or the flowchart-based approach by McGraw and Wong (21) in that by impartially using and comparing the results of the three single-score ICC formulas, the presence of bias can be evaluated. Recently, a method for the estimation of intra-cluster correlation for clustered categorical data has been published (22).

### The Svensson method

Characteristics of the Svensson method include the following: data type = paired ordered categorical data; number of raters = two; analysis software = R package svenssonm, downloadable Excel sheet; estimate to report = several different scores; significance analysis = 95% CI, *P* value; pros = handles ordinal data well; cons = difficult to calculate manually.

Primarily, the Svensson method is used to examine change in ordinal data (e.g. pain effect after intervention). The method (11,12) is based on ranking methods; hence, the score is ordinal for two raters or paired observations. The systematic disagreement of the marginal distributions is described using four measurements: relative concentration (RC); relative position (RP); augmented rank-order agreement coefficient (r_a_); and individual variation (RV). RC measures the systematic difference in concentrations of scores (i.e. how well the raters assess the items in a similar way), RP measures the systematic shift in positions between pairs (either over- or underestimation), and RP measures any systematic shift in scoring between raters. Both RC and RP are in the range of −1 to 1, with 0 indicating no systematic disagreement. The unit is percentage points (percent units). Hence, values of RP and RC close to zero indicate a high inter-rater reliability.

The augmented rank-order agreement coefficient (r_a_) is a correlation of the pairs of augmented mean rank values and measures. The individual variation, RV, is in the range of 0–1, with 0 indicating no random contribution. A high value of r_a_ and a low value of RV indicate minimal individual dispersion from the rank-transformable pattern. It is quite complicated to calculate this by hand, but there is a package for the R statistical software and an Excel sheet (23) (available for download where up to 11 categories can be measured). A practical example is given in the study by Vult von Steyern et al. (24) where inter-rater reliability is measured between two raters for a scoring system for tomosynthesis in pulmonary cystic fibrosis.

### Kappa statistics

#### Cohen's kappa

The characteristics of Cohen’s kappa include the following: data type = nominal or ordinal; number of raters= two; analysis software = standard statistical packages; estimate to report = a value between −1 and 1, where ≤0 indicates absence of reliability; significance analysis = 95% CI; pros = easy to calculate and commonly used; cons = highly sensitive to prevalence, skewed data, and sample size. Kappa values cannot be compared reliably across studies

Cohen's kappa (25) is probably the most popular variant of the kappa statistic (see the example given in Geijer et al. (26)) and can be used unweighted or with linear or quadratic weights (27,28). A deeper theoretical discussion can be found in the paper by Hallgren (6). With weighting, the statistic takes not only agreement but also association into account (29). The numerical results are usually translated into words (poor, slight, fair, moderate, substantial, and almost perfect) according to Landis and Koch (30). There are several limitations to Cohen's kappa, such as being limited to two raters and having applicability only for categorical data. Cohen's kappa is influenced by the distribution of data (31), and thus by the number of chosen categories, and by a high or low true prevalence (28). If the true prevalence of a population is high or low, the agreement expected by chance increases, and the magnitude of kappa declines (32). As the statistic is heavily influenced by the nature of the data, it is impossible to compare the kappa value between studies or rank the rater agreement, other than in broad terms.

#### Agreement coefficients by Gwet

The characteristics of Gwet include the following: data type = both nominal (AC1) and ordinal or quantitative scaled (AC2); number of raters = two or more; analysis software = R statistical package; estimate to report = a value between −1 and 1, where ≤0 indicates absence of reliability; significance analysis = 95% CI; pros = less sensitive to prevalence and skewed data than Kappa statistics; cons = Gwet's AC1 and Kappa values cannot be compared, thus the translation of Kappa values by Landis and Koch (30) is inappropriate for Gwet's AC1.

It is well known (33) that Kappa statistics might give an inconsistent value if there is an imbalance in the marginal totals, such as an imbalance in prevalence of the patients with a diagnosis (e.g. the illness is rare). For instance, even if the percent agreement is high, but the disease is rare, the Kappa value might be low. This is a highly contradictory result and is denoted “the Kappa paradox” (33).

An alternative measure is to use the Gwet AC1 statistic. Gwet also developed a weighted version of the AC1-statistic, appropriate for ordinal and interval scaled data, which is called Gwet´s AC2 (8,10). The calculation of Kappa and Gwet's AC1 statistic are very similar at a first glance. Both methods calculate the difference between the observed agreement and the chance of agreement, divided by the complement of the chance of agreement. The two measurements differ in how the chance of agreement is calculated. Kappa calculates the probability using both raters’ scoring of each patient as healthy or sick similarly, independently of each other. Gwet's AC statistic, on the other hand, bases the chance of agreement calculation on the probability of the raters performing a random rating and agreeing, using a conditional probability. If the prevalence for a positive rating is the same for the two raters, it can be shown that Gwet's AC statistic uses the rate of disagreement instead of the rate of agreement, as in the Kappa statistic (34).

When the prevalence is 50%, the Kappa statistic and Gwet's AC1 coincide; however, when the prevalence differs, Gwet's AC1 gives a value more similar to the percentage agreement. Still, the interpretation of the two values differs and it has been questioned if the results really can be compared (34).

#### Fleiss’ kappa

The characteristics for Fleiss’ kappa include the following: data type = nominal; number of raters = two or more; analysis software = standard statistical packages; estimate to report = a value between −1 and 1, where ≤0 indicates the absence of reliability; significance analysis = 95% CI; pros = addresses multiple items and multiple raters; cons = unsuitable if missing data or higher than nominal order. Confidence intervals should be calculated by bootstrapping.

Fleiss’ kappa (35,36) measures agreement between multiple items and multiple raters. The outcome should be nominal (Fleiss’ kappa ignores the order if the outcome is ordinal) and all raters should use the same categories for multiple readers (35). Fleiss’ kappa reduces to Scott's π (a predecessor to Cohen's kappa) when having two raters. The raters should be non-unique (37); hence, the group of raters is randomly chosen from a larger population. The method is also limited to balanced data, where each object is scored by the same number of raters and is vulnerable to missing observations. Fleiss’ kappa is unsuitable if there are missing data and for higher than nominal order, where Krippendorff's alpha is a better alternative (38). Asymptotic confidence intervals should not be used, instead bootstrapping using an R script provided by Zapf et al. (38) is suggested. Another alternative is multi-rater kappa where variants exist (39,40), which solves the problem associated with applying fixed-marginal kappa (e.g. Cohen's kappa and Fleiss’ kappa) to free-marginal distributions. The differences and similarities of different multi-rater kappa variants have been discussed by Warrens (41). When using variants of the kappa statistic, the same dataset may result in different kappa values (41). Modern-day model-based techniques are available to evaluate rater agreement between multiple raters (29).

#### Krippendorff's alpha

The characteristics for Krippendorff’s alpha include the following: data type = all scales; number of raters = two or more; analysis software = statistical packages or Internet calculator; estimate to report = a value between −1 and 1, where ≤0 indicates absence of reliability; significance analysis = bootstrapping gives a *P* value or confidence intervals; pros = handles all types of data and incomplete data; cons = does not distinguish between systematic bias and random effect.

Krippendorff's alpha (4) gives a measurement in the range of −1 to 1, where 0 denotes the absence of reliability, −1 indicates a high level of disagreement, and 1 indicates perfect reliability. The method can be used for all different scales of outcome. The advantages of Krippendorff's alpha are that the method can handle incomplete datasets, different sample sizes and numbers of raters, and all types of data (nominal, ordinal, or quantitative outcome). However, the calculations are complex and, like other kappa measures, it does not distinguish between systematic bias and random effect. According to Hayes and Krippendorff (4), for the two-rater case with a nominal outcome, α coincides asymptotically with Scott's π, and for the two-rater case with an ordinal outcome, α is the same as Spearman's rank correlation coefficient ρ (rho; without ties in ranks). Finally, in the two-rater case with an interval outcome, α equals Pearson et al.’s ICC (42). An R script for calculating Krippendorff's alpha and bootstrapping confidence intervals is provided by Zapf et al. (38). An Internet calculator for Krippendorff's alpha is also available (43,44).

## Other methods used in rater agreement analysis

### Visual grading characteristics (VGC)

The characteristics of VGC include the following: data type = ordinal; number of raters = two or more; analysis software: custom, freely available; estimate to report = area under the curve (AUC) value; significance analysis = *P* value, 95% CI – given by bootstrapping; pros = handles ordinal data without the need for agreement on scoring steps between raters; cons = custom software required.

VGC is a method primarily developed for analyzing visual-grading data that result from raters grading perceived image quality (13). VGC may be used to assess the visibility of a structure, the sharpness of an anatomic structure, or the possibility of discriminating one structure from another. The method often uses a standard set of criteria, such as the European Commission guidelines (45–47) and an ordinal rating scale of typically 4–5 scale steps. In VGC analysis, the raters thus may score an item in the range of 1–5 (poor sharpness to perfect sharpness) on two sets of images, e.g. from 1.5-T and 3-T MR images. The rating data for these two sets of images are then used to determine a so-called VGC curve, which describes the relationship between the cumulative ratings for the two sets of images. The AUC is used as an overall figure-of-merit in the analysis. An AUC statistically separated from 0.5 indicates a statistically significant difference in image quality between the two sets of images. The method handles single and multiple raters and paired and non-paired data. VGC is not a method for evaluating rater reliability per se, but rater reliability analysis is a built-in feature of the method that uses bootstrapping and specialized software. The built-in assessment of both intra- and inter-rater reliability influences the 95% CI of the reported AUC and the *P* value (14,48).

The analysis of the rater agreement in VGC analysis is not based on the assumption that the raters interpret the rating scale in the same way. Further suggestions on the application and interpretation of VGC results have been published recently (14). However, a variant of VGC analysis may be used to analyze the inter-rater reliability between two raters. In this variant, a VGC curve is determined that describes the relationship between the cumulative scores of two raters. Here, a VGC curve close to the diagonal indicates high inter-rater reliability, whereas an AUC statistically separated from 0.5 indicates a statistically significant difference between the two sets of ratings. However, an AUC that is not statistically separated from 0.5 cannot be taken as a sign of no difference between the sets of ratings. An AUC of 0.5 can be also obtained when the VGC curve deviates from the diagonal. This use of VGC analysis bears a strong resemblance to the Svensson method, although the single scalar AUC cannot describe the relation between the two raters to the same extent as the four measurements used in the Svensson method.

### Visual grading regression (VGR)

The characteristics of VGR include the following: data type = ordinal; number of raters = two or more; analysis software = standard statistical packages; pros = allows for the assessment of several items in the same experiment; cons = does not analyze rater reliability.

VGR applies ordinal logistic regression to ordinal data from image quality scoring of single-image or image-pair experiments (15). It allows for several items to be assessed in the same experiment, such as images from different CT scanners using several reconstruction algorithms with several dose levels, a situation where VGC analysis would be limited to assessing one item at a time. Rater reliability is, however, not analyzed separately using this method. Both patients and raters are seen as samples from a larger population and treated as random effects, which can also be done with ordinal regression models (49). Thus, other methods described in this article must be used to assess rater agreement. The calculations of VGR are complex and further information on ordinal logistic regression can be found in standard textbooks on statistics (50).

### Consensus scoring

The pros of consensus scoring include that it is easily calculated, while the cons are it is not a statistical method.

Consensus scoring (16) is not a statistical method per se, but a method to obtain rapidly a scoring from two or more raters. The method is practical and useful in many situations but is flawed by the lack of statistical analysis and inherent bias of various types, not least by the possible larger influence by the more experienced rater over the less experienced. Consensus scoring may be performed in several ways, and it is important to report how the scoring was performed (16).

### Percent agreement

The pros of percent agreement include that it is easily calculated. The cons are that it overestimates agreement since agreement by chance is disregarded and does not consider the amount of disagreement.

Percent agreement is the proportion in which two raters report the same scores of nominal data. It applies only to two raters and can, according to Hayes and Krippendorff (4), only give a correct evaluation if the agreement is perfect, i.e. 100%. An agreement of 0%, for example, is implausible from observations made by independent raters since that would require disagreement on every observation (4). It does not take agreement by chance into account, thereby overestimating agreement (6), or consider the amount of disagreement, thus being sensitive to the number of categories.

## Correlation measures

**Fig. 1.**
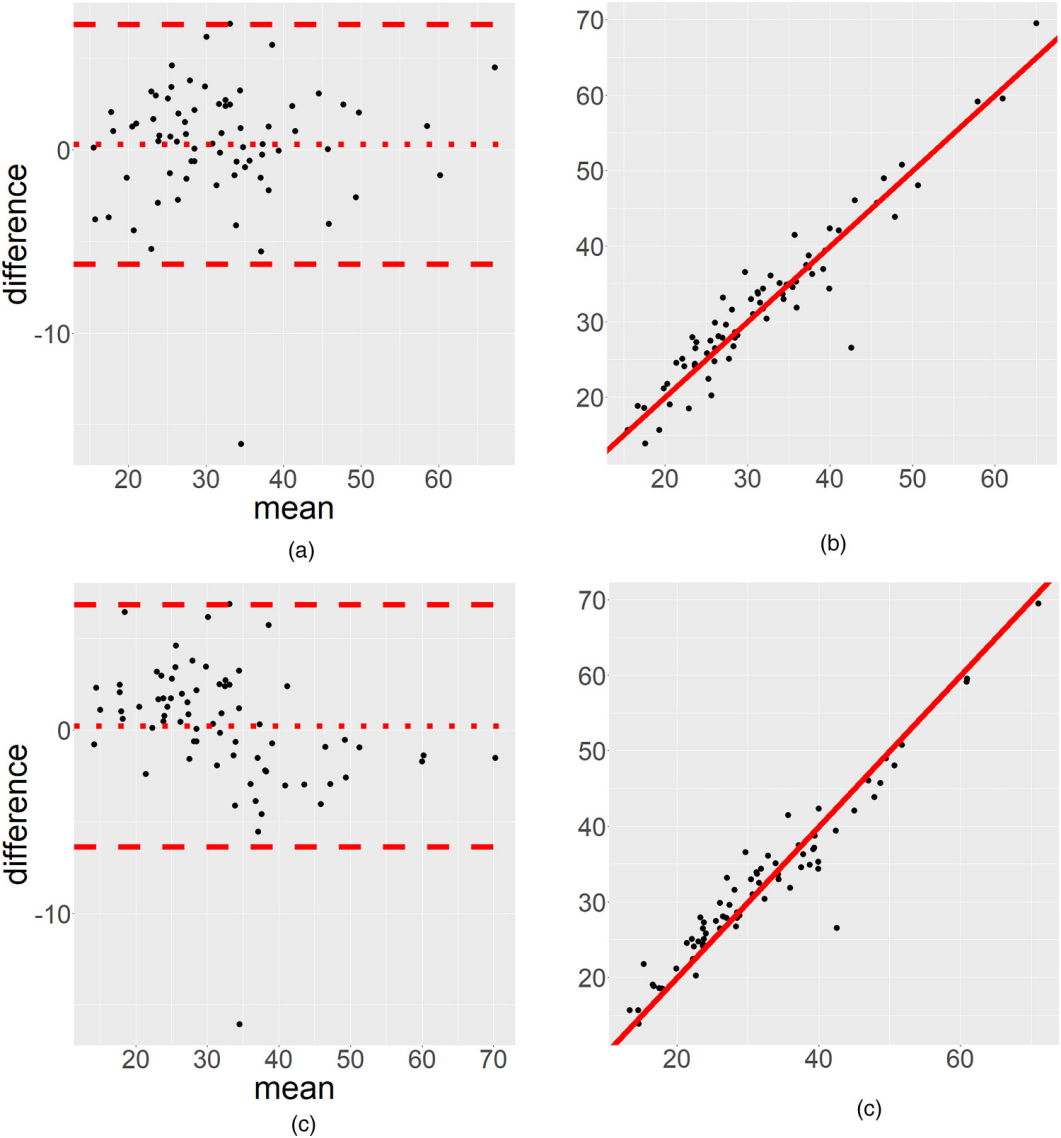
We have used data from Geijer et al. (51) to illustrate the advantage of (a) a Bland–Altman plot compared to (b) a scatterplot. (c, d) Various aspects of Bland–Altman plots on slightly modified data. (a) A small, ignorable systematic difference, with a comparable high random effect, hence the Bland–Altman plot makes it possible to distinguish between a systematic bias and random effects. (b) The scatter plot (on the same dataset) cannot show this distinction (the Spearman correlation is 0.93). (c) We analyze a modified version of the same data, where the Bland–Altman plot indicates an overestimation for low values and an underestimation for high values between the two raters. (d) This is not obvious when interpreting the same data in the scatterplot (the Spearman correlation coefficient is 0.94).

A correlation measure is sometimes used to compare the scoring between two raters. However, the correlation may be perfect (close to 1) despite a low rater agreement (17) (Fig. 1). Thus, a correlation should not be used in place of established statistical methods for rater agreement analysis.

### Bland–Altman plots

A graphical presentation may be used to display the relationship between the two raters’ scores. A common way to illustrate agreement but with limited information is to show a scatterplot displaying the relation between the two raters’ scores (Fig. 1), often departing from the 45° line. The scatterplot may be misleading and indicate a high correlation, which is not the same as a high level of agreement, but does not show if there is a bias for low or high values. We strongly discourage readers from using this illustration (17). Instead, we recommend using a Bland–Altman plot for continuous data (Fig. 1). A Bland–Altman plot can illustrate if a disagreement occurs for low or high values (17) and is thus an important visual complement to the numerical statistical rater agreement analysis. Taffé et al. (52) have highlighted some serious concerns regarding the assumptions behind the Bland–Altman plot when the data do not have proportional bias. This becomes a problem when the plot is interpreted at a population level. We therefore recommend the use of Bland–Altman plots as a descriptive measure only.

## Conclusion: selection of an appropriate method for rater agreement analysis

Common features of kappa estimates (i.e. Cohen’s, Fleiss’, Gwet’s AC1, and Krippendorff´s) are that these methods give an estimate, representing rater agreement, between −1 and 1. These estimates have been criticized as ambiguous and difficult to interpret (27). On the other hand, the Svensson method, ICC, and Bland–Altman plots (the latter is not a strict test at a population level, but a useful graphical and descriptive tool) distinguish between a random effect and a systematic effect.

Assessment of rater agreement is an important and integral part of image quality studies in radiology, and depending on the outcome of the assessment, selecting the appropriate method from an available toolbox of statistical analysis methods is important. In Table 4, some suitable methods have been suggested. Special conditions may mandate that other methods be used. The selection and further statistical analysis require different approaches depending on the data type, the structure of the data, and the number of raters. Often, it is informative to use multiple methods to truly understand the effect. This brief report cannot give a full description of the methods and the reader needs to be aware that the measures describe different aspects of the data.
